# Pulsed electromagnetic fields for post-appendicectomy pain management: a randomized, placebo-controlled trial

**DOI:** 10.1186/s13063-022-06810-y

**Published:** 2022-10-14

**Authors:** Muralitharan Perumal, Aizatul Isla Abdul Latib, Malarvilee Paul Samy, Mohd Rohisham bin Zainal Abidin, Elanngovan Nagandran, Tham Sim Wan, Pamela Francis, Chee Yoong Foo

**Affiliations:** 1 Department of Anesthesiology, Hospital Tengku Ampuan Rahimah, Klang, Malaysia; 2 Clinical Research Centre, Hospital Tengku Ampuan Rahimah, Klang, Malaysia; 3 National Clinical Research Centre, Shah Alam, Malaysia; 4 IQVIA Asia Pacific, Petaling Jaya, Malaysia

**Keywords:** Pulsed electromagnetic fields, Postoperative pain, Appendicectomy, Randomized trial

## Abstract

**Background:**

The value of pulsed electromagnetic field (PEMF) in postoperative pain management, due to the inconsistent findings so far, remains unclear. We extended the evaluation of PEMF on postoperative pain and intravenous (IV) analgesic use to a group of post-appendicectomy Asian patients.

**Methods:**

This is a double-blinded, randomized trial. Adults with a clinical diagnosis of acute appendicitis were enrolled. Patients were allocated randomly to receive an active-PEMF device or an inactive device after the surgery in addition to the standard postoperative pain management. The primary outcome measure was the 12-h cumulative postoperative pain intensity measured at four different time points using the visual analogue scale. The secondary outcome measure was the total amount of IV fentanyl used (in mg) via PCA over the first 12 postoperative hours. The primary analysis in this trial compared the two study groups for the reported cumulative pain score (both at rest and on movement) and the cumulative amount of IV fentanyl uses over the first 12 postoperative hours using the Wilcoxon rank sum test. Analyses were performed based on the intention-to-treat principal. Multiple imputation was used to handle the missing data assuming that the data were missing at random.

**Findings:**

One hundred eighteen subjects were randomized; 58 were allocated to the active-PEMF group and 60 to the inactive control group. Pooled mean pain score of both intervention groups by time point declined in a similar fashion over the course of 12 postoperative hours. The 12-h cumulative postoperative pain score at rest and on movement did not differ significantly after the procedure. (*W* = 1832.5 ~ 1933.0, *p*-value 0.6192 ~ 0.2985 for resting pain score comparison; *W* = 1737.0 ~ 1804.5, *p*-value 0.9892 ~ 0.7296 for movement pain score comparison). For the secondary outcome measure of 12-h total fentanyl use, a comparison between the PEMF vs. placebo arm also revealed no statistically significant difference across all the 20 imputed datasets (*W* = 1676.5 ~ 1859.0, *p*-value 0.7344 ~ 0.5234).

**Discussion:**

PEMF was not superior to placebo as an adjunct pain management for up to 12 h post-appendicectomy. Previously reported effect of PEMF on postoperative pain intensity and analgesia uses in similar surgical settings cannot be verified.

**Trial registration:**

National Medical Research Register Malaysia (NMRR-15–670-25,805) and Thai Clinical Trials Registry (retrospectively registered on November 01, 2019, Study ID—TCTR20191102002).

**Supplementary Information:**

The online version contains supplementary material available at 10.1186/s13063-022-06810-y.

## Introduction

Acute appendicitis is one of the most common indications for surgery in patients admitted to the hospital for acute abdominal pain [1, 2]. Urgent appendicectomy is still the recommended treatment for acute uncomplicated appendicitis [3, 4]. Although emergency appendicectomy is usually well tolerated by most patients, postoperative pain is nevertheless an important postoperative morbidity. It prolongs hospital stay, increases healthcare costs and results in higher risks of various long-term adverse outcomes [5]. Currently available methods of postoperative pain management have undesirable adverse effects and their effectiveness varies across individuals [6, 7]. Epidural analgesia, a highly effective postoperative pain control approach, requires technical expertise that might not be readily available, especially in a setting of limited resources. It also has a number of contraindications and can potentially result in complications [8]. Opioid use is similarly associated with various side effects [9]. Hence, a safer and more effective post-appendicectomy pain management approach is still highly sought after.

Evidence suggests that pulsed electromagnetic field (PEMF) when applied to injured tissues has the properties of increasing the release of anti-inflammatory cytokines, endogenous analgesic agents, and opioid precursor proteins in these tissues as well as reducing pro-inflammatory cytokines and endogenous hyperalgesia agents, thereby reducing pain and improving tissue healing [10, 11].{Rohde, 2010 #262;Chua, 2011 #264;Hagiwara, 2009 #265} Over the past decades, researchers have tried PEMF for postoperative pain management under different surgical scenarios, particularly on plastic and oral surgeries [12–15]. Unfortunately, the results were not sufficiently consistent. For example, Heden and Pilla (2008) [12] reported a controlled trial of PEMF use among 42 females with post-breast augmentation surgery and found a large and significant effect (more than 3 times different). More recently, Khooshideh et al. (2017) reported a placebo-controlled study of PEMF on postoperative pain, analgesia uses and wound healing in 72 patients undergoing caesarean section [16]. The study found that patient-reported pain score and analgesia uses were significantly lower among those assigned to PEMF vs. the placebo control. On the other hand, Stocchero and colleagues in 2014 [15] showed negative findings after comparing similar measures of pain score and analgesia use among those who have undergone unilateral mandibular third molar extraction via a randomized blinded study. Similar negative results have also been reported in a number of controlled studies: post blepharoplasty [17], breast augmentation [18] and dental implantation procedure [14].

The value of PEMF on postoperative pain management, due to the inconsistent findings so far, remains unclear. In this study, we extended the evaluation of PEMF on postoperative pain and intravenous (IV) analgesic use to a group of post-appendicectomy Asian patients. We hypothesized that, if PEMF therapy does improve postoperative pain in appendicectomy, IV fentanyl use via PCA is expected to reduce while the perceived postoperative pain will at least remain (if not reduced concurrently). The logical framework of the study hypothesis is provided as Supplementary Material (1).

## Materials and methods

### Patients

We undertook a prospective, randomized, double-blinded, placebo-controlled trial. The study took place in the Hospital Tengku Ampuan Rahimah, Klang (a tertiary referral centre) Malaysia. The study protocol was reviewed by and registered with the National Medical Research Register, Malaysia (NMRR-15–670-25,805) prior to the enrolment of the first subject. Additional registration of the study protocol to the Thai Clinical Trials Registry was also performed retrospectively (Study ID: TCTR20191102002). All patients provided signed informed consent. The trial started its recruitment on 1st August 2015 and ended on 31st December 2015 for accomplishing the intended sample size.

All adults examined in the emergency department and suspected to have acute appendicitis were assessed for possible inclusion in the study. Patients were excluded if one of the following criteria were present: age less than 19 years or more than 50, pregnancy or a positive pregnancy test, had underlying chronic pain or history of long-term opioid use, appendicectomy lasted more than 3 h or inability to understand information about the protocol or to sign the informed consent form. The on-duty medical officers from the anaesthesiology department enrolled the eligible patients. Patients eligible for inclusion to the study were informed of the protocol and invited to participate.

### Randomization and masking

After informed consent was obtained, patients were individually and randomly assigned to either the active PEMF group or the control group (inactive PEMF) according to the randomization schedule prepared prior to the trial initiation. A simple randomization with a 1:1 allocation ratio in this study. The on-call medical officer taking care of the postoperative recovery room performed the random assignment (by opening a consecutively numbered envelope that contained the random allocation code) before the operation and performed the placement of the trial device onto the enrolled subjects after the operation. Opaque, sealed, identical and sequentially numbered envelopes (SNOSE) were used.

The randomization schedule (with the allocation codes) was generated by a trial researcher not involved in the care of the trial subjects prior to the trial start. The same researcher also prepared the numbered envelopes according to the computer-generated schedule. A free, web-based randomization schedule generator from www.graphpad.com/quickcalcs/randomize1/ was used.

### Procedures

**Fig. 1:**
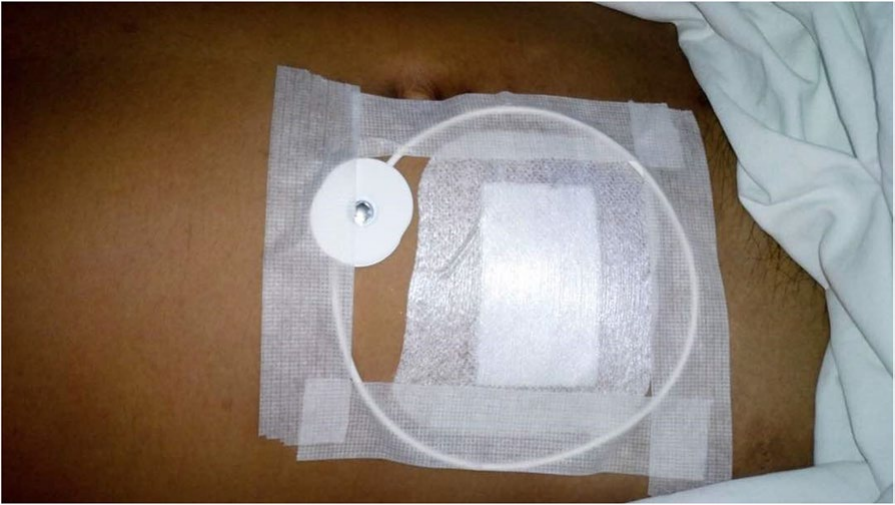
PEMF device placed on the surgical wound on top of the dressing for 12 h

Immediately after the appendicectomy, either an active or an inactive PEMF device was placed on the surgical wound on top of the dressing for 12 h (see Fig. 1). Patients were admitted to the hospital after the operation irrespective of the trial intervention received.

The PEMF device used in this study (RecoveryRx, BioElectronics Corp.) is a battery-powered 12-cm elliptical coil radiofrequency energy generator (frequency = 27.1 MHz, pulse rate = 1000 per second, pulse duration = 100 ms). The surface of the device was cleaned and disinfected using 70% isopropyl alcohol wipes prior to application. All visible soil (if any) were first removed via the first wipe. A second wipe was then performed to disinfect the device’s surface area. The wipe contacted the whole device surface for at least 3 s during the disinfection process.

Appendicectomy was performed according to surgeons’ standard practice (via a McBurney incision). All patients also received general anaesthesia according to the standard protocol. Anaesthesia was induced with IV fentanyl (1–1.5 μg/kg to a maximum of 100 μg) and propofol (2–3 mg/kg), followed by a muscle relaxant of the anaesthesiologists’ choice. All patients then receive IV morphine 0.05 mg/kg intraoperatively. Surgery was commenced after the patient was successfully anaesthetized. No local infiltration of any anaesthetic agents was performed on the incised skin. All patients were supplemented with patient-controlled analgesia (PCA) with IV fentanyl postoperatively for 12 h.

### Assessments and outcome measures

The primary outcome measure of this study was the 12-h cumulative postoperative pain intensity measured at four different time points using the visual analogue scale (VAS). The secondary outcome measure was the total amount of IV fentanyl used (in mg) via PCA over the first 12 postoperative hours. Participants were asked to report their pain intensity based on the VAS. Pain intensity was evaluated at 0, 4, 8, and 12 h after surgery. The requirement for PCA IV fentanyl postoperatively was recorded by a research assistant.

The VAS is a unidimensional measure of pain intensity [19]. It is a continuous scale comprised of a horizontal line of 10 cm in length. The scale ranges from 0 (representing no pain) to 10 (representing worst imaginable pain). Patients were educated on how to use and report their pain with the VAS. They were also asked to place a crossline on the unmarked horizontal scale at the required times interval.

PCA is a pain control system which allows a patient to self-administer a predetermined dose of analgesic medication for pain relief [20]. Fentanyl is a mu-opioid receptor agonist with strong analgesic effects, rapid onset of action and a low risk of adverse effects [21]. IV-PCA using fentanyl has been widely adopted globally for postoperative pain management. We used the [CADD Legacy PCA, model 6300; Smiths Medical International, Kent, UK] PCA system in this study. The PCA system was programmed as follows: bolus, 1 mL; lockout time, 5 min; maximum dose, 6 mL/h; no background infusion. Infusion solutions containing 1.0 mg of fentanyl were adjusted to 100 mL by dilution with saline (concentration of fentanyl, 10 μg/mL). The amount of IV fentanyl used (in mg/dL) via the PCA system was recorded over the first 12 h after the operation. It is expected that, if the PEMF therapy is effective in reducing postoperative pain, lower amount of IV fentanyl would have been used via the PCA.

The patients and the surgeons, nurses and other healthcare workers including the acute pain service (APS) team involved with direct patient care were blinded to the allocation group. The APS team provided all pain-related clinical management to all study subjects. The research assistant who collected the PCA data was also blinded to the allocation. The identification of treatment was concealed by using an identical yet inactive device.

### Statistical analysis

This study was based on the notion that PEMF therapy would reduce postoperative pain and analgesia use in appendicectomy. We estimated the required sample size based on the Wilcoxon rank sum test using the Monte-Carlo simulation technique. In the simulation, we assumed that the mean cumulative pain score in the control group vs. the intervention was 1.0 and 2.0 respectively. The expected difference of cumulative VAS pain score was 1.0. [19]. A common standard deviation of 1.5 (for the pain score) for both groups was used [16]. The sample size was then varied by simulation until the proportion of significance (0.05) reaches the desired power of 0.80. Thereafter, an additional 10% dropout rate was added to the target sample size. The simulation was performed using the MKpower package (https://cran.r-project.org/package=MKpower).

Based on the simulation, we found that a sample size of 80 patients (in the ratio of 1:1 for intervention and control group) would give an 80% power to establish whether PEMF treatment was superior to placebo in relation to postoperative pain reduction. With the additional consideration of potential dropout (10%) and rounding to the nearest full number, we determined that the target sample size should be 100 (50:50 for control and intervention).

To assess the hypothesized effect of PEMF on postoperative pain, we compared the two study groups for their reported cumulative pain score (both at rest and on movement) and the cumulative amount of IV fentanyl uses over the first 12 postoperative hours using the area-under-the-curve (AUC) approach. For each subject, the AUC for pain scores and IV fentanyl uses were calculated by summing their values over the12 postoperative hours. The distribution of AUC for pain score and IV fentanyl uses between the two study groups were compared using the Wilcoxon rank sum test with continuity correction. Analyses were performed based on the intention-to-treat (ITT) principal. All reported p values are two-sided and were not adjusted for multiple testing. We used R version 3.3.2 for all the analyses.

There were 21 subjects (17.8%) with at least one missing data on the primary outcome measure (pain score on one of the 4 time points) and 28 subjects (23.7%) with at least one missing data on the secondary outcome measure (IV fentanyl use on one of the 3 time points). Multiple imputation by chained equations from the *mice* package in R was used to handle the missing data assuming that the data were missing at random. Baseline variables (including age, sex, BMI and race), trial variables (intervention group and observational time) and outcome variables (pain score and fentanyl use at each time point) were used to form the imputation models. Predictive mean matching with auto-generation of the predictor matrixes was used. Twenty imputed datasets were generated for subsequent analyses. For visual assessment, the mean pain scores and IV fentanyl uses for each time point across the imputed datasets were pooled according to the Rubin rules and plotted on a line graph. For results of the Wilcoxon rank sum test, pooling was not done due to the expected non-normal distribution and small sample size. Instead, we directly reported the test statistics (*W*) and *p*-values of each analysis performed on the 20 imputed datasets and summarized them by range.

To assess the stability of the conclusion to the missing-at-random assumption, a best-case and worst-case sensitivity analysis was undertaken. Under the best-case analysis, all PEMF subjects with missing data were assigned with the 1st quartile value of pain score and IV fentanyl use from the PEMF subjects with observed data of the same time point. All placebo subjects with missing data were assigned with the median value of pain score and IV fentanyl use using the same approach. Under the worst-case analysis, missing data were imputed in the reverse fashion using 3rd quartile value for PEMF subjects and the median value for the placebo subjects.

## Results

**Fig. 2:**
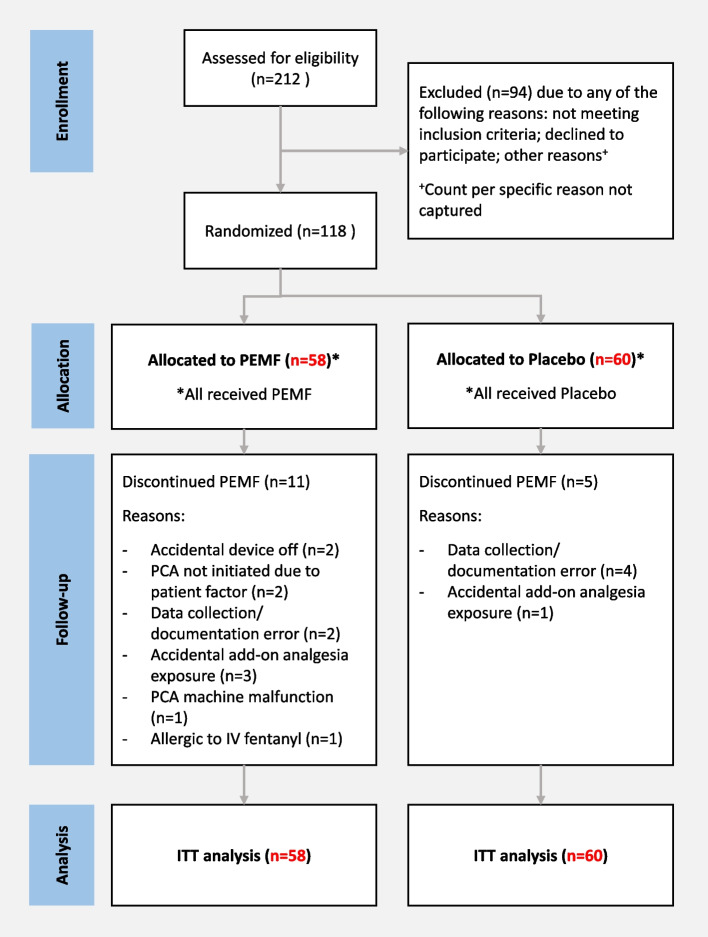
Consort diagram

A total of 118 subjects were enrolled and randomized: 58 were assigned to the active-PEMF group and 60 to the inactive PEMF (control) group. 11 subjects from the intervention group and 5 subjects from the control group discontinued the assigned treatment before study completion due to the following reasons: accidental device off, PCA not initiated due to patient factor, data collection/documentation error, accidental add-on analgesia exposure, PCA machine malfunction, and allergic to IV fentanyl. Ultimately, 47 subjects from the intervention group and 55 subjects from the control group completed the study per protocol. Per the ITT principal, all assigned subjects were included for analysis of primary and secondary outcome measures (see Fig. 2).

Table 1 shows the baseline characteristics of the trial participants and the comparison of these characteristics between the intervention and placebo groups. Overall, these characteristics appear balance between the intervention group and the placebo group. The baseline (average) pain scores at rest and on movement were also similar between the two comparison groups (see Table 1).

**Table 1:** Demographics and baseline characteristics of participants

	Overall	Placebo	PEMF	*P*-value
Number of patients	118	60	58	
Age (mean (SD))	27.5 (7.2)	27.1 (6.9)	28.0 (7.6)	0.462
Sex = Male (%)	79 (66.9)	38 (65.5)	41 (68.3)	0.897
BMI (mean (SD))	22.9 (4.40)	23.0 (4.66)	22.8 (4.15)	0.878
Ethnicity (%)				0.437
Indian	19 (16.1)	7 (11.7)	12 (20.7)	
Malay	64 (54.2)	33 (55.0)	31 (53.4)	
Non-citizen	23 (19.5)	12 (20.0)	11 (19.0)	
Others	12 (10.2)	8 (13.3)	4 (6.9)	
Perforated appendicitis (%)	12 (11.8)	9 (16.4)	3 (6.4)	0.211
**Baseline readings**				
Systolic BP (mean (SD))	124.6 (12.3)	123.9 (12.4)	125.4 (12.1)	0.313
Pulse rate (mean (SD))	73.5 (12.5)	73.1 (12.5)	74.0 (12.6)	0.866
Respiratory rate (mean (SD))	16.7 (3.1)	16.9 (3.1)	16.5 (3.0)	0.725
Postoperative resting pain score (mean (SD))	2.5 (1.2)	2.4 (1.1)^a^	2.6 (1.4)	0.617
Postoperative pain score on movement (mean (SD))	3.5 (1.3)	3.5 (1.2)^a^	3.6 (1.4)	0.678

^a^Two subjects had missing data for this variable

**Fig. 3:**
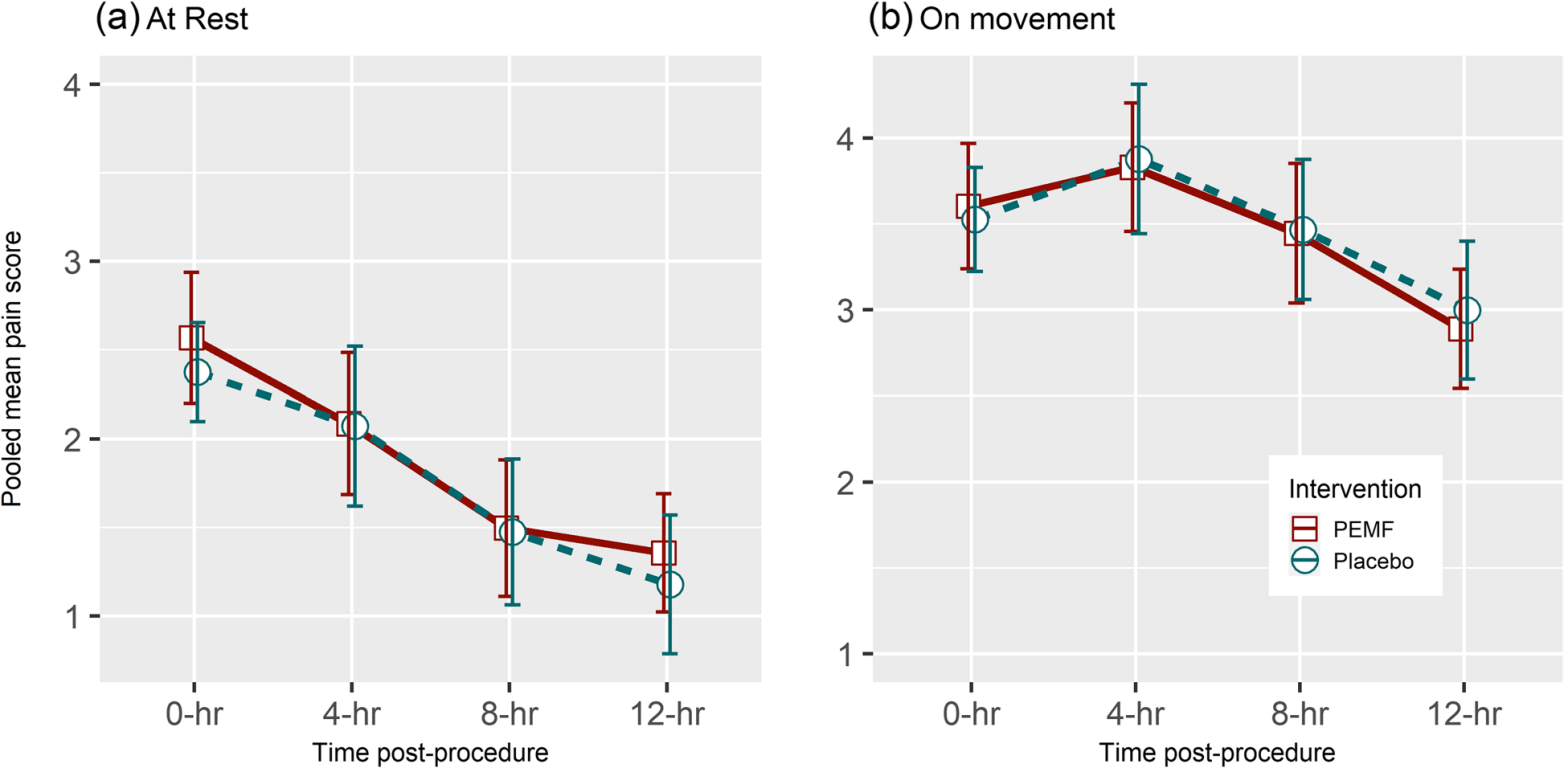
Changes of pain score post-appendectomy—PEMF (*n* = 58) vs control (*n* = 60). A higher pain score means more pain. An annotated version of the same graph depicting the number of subjects with complete data at each observed time point by intervention group is provided in Supplementary Material 6

Pooled mean pain score of both intervention and placebo groups by time point declined in a similar fashion over the course of 12 postoperative hours (Fig. 3). The pooled mean pain scores at the end of the observation period (i.e. 12 h after the operation) were 1.4 (SD = 1.3) for the PEMF group and 1.2 (SD = 1.5) for the placebo group at rest. On movement, it was 2.9 (SD = 1.3) for the PEMF group and 3.0 (SD = 1.5) for the placebo group. The change pattern appears similar for both the resting pain score and pain score on movement (Fig. 3). Comparison of the AUC for pain score (at rest and on movement) between the PEMF vs. placebo arm using the Wilcoxon rank sum test showed no statistically significant difference across all the 20 imputed datasets (*W* = 1832.5 ~ 1933.0, *p*-value 0.6192 ~ 0.2985 for resting pain score comparison; *W* = 1737.0 ~ 1804.5, *p*-value 0.9892 ~ 0.7296 for movement pain score comparison; see Supplementary Material 2). Sensitivity analysis using the best-case-worst-case approach revealed similar results to the above described (details of the sensitivity analysis are available in Supplementary Material 3).

**Fig. 4:**
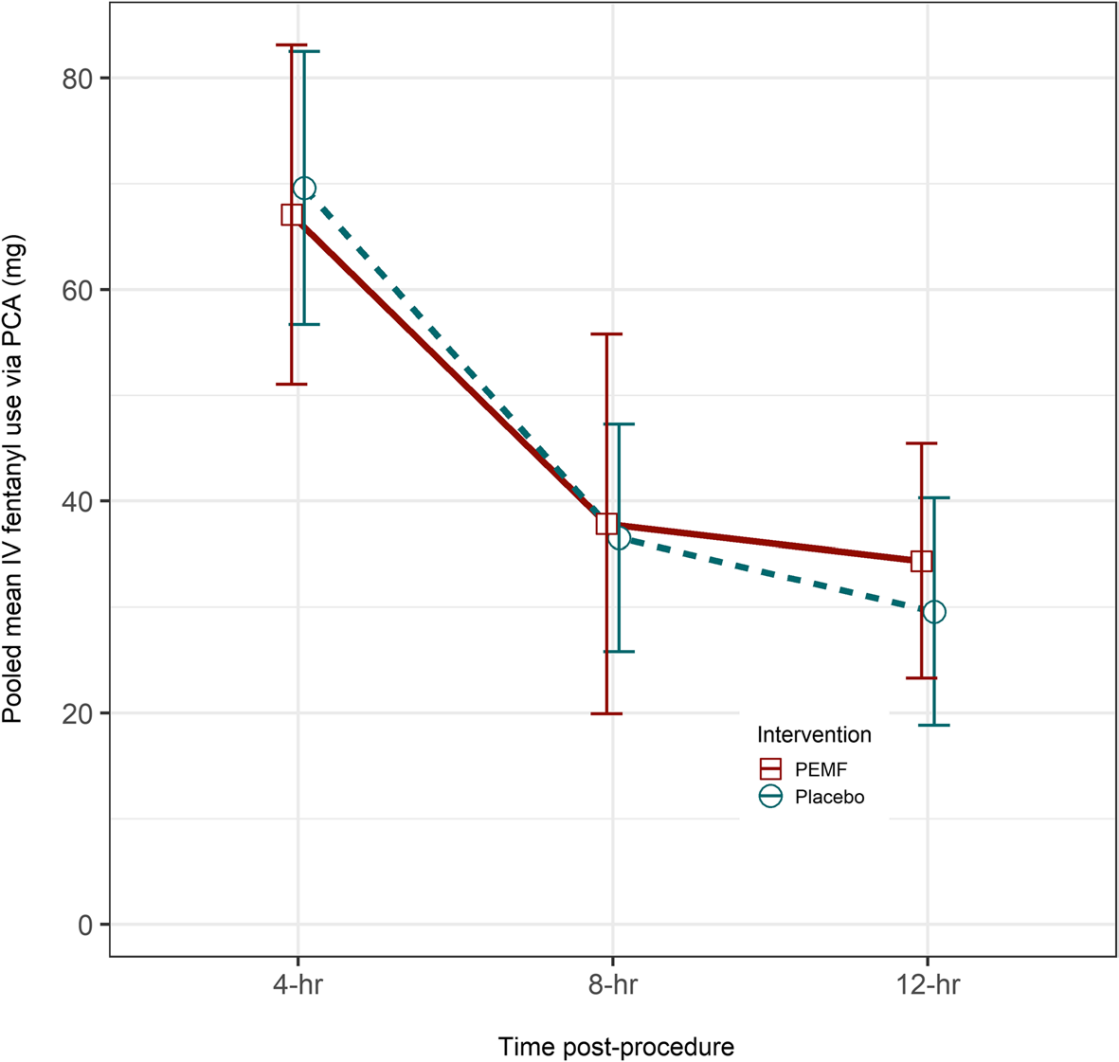
Changes of PCA fentanly use post-appendectomy—PEMF (*n* = 58) vs control (*n* = 60). An annotated version of the same graph depicting the number of subjects with complete data at each observed time point by intervention group is provided in Supplementary Material 6

For the secondary outcome measure of 12-h total fentanyl use, a comparison between the PEMF vs. placebo arm also revealed no statistically significant difference across all the 20 imputed datasets (*W* = 1676.5 ~ 1859.0, *p*-value 0.7344 ~ 0.5234; see Supplementary Material 4). The utilization patterns show similarity between both arms using the pooled mean of each time point (Fig. 4). Conclusion was not affected by the best-case-worst-case sensitivity analysis (see Supplementary Material 5).

## Discussion

This randomized controlled study involving 118 post-appendicectomy patients indicates no significant effect PEMF has on both the patient-reported pain intensity measures (i.e. resting and moving) over the initial 12 h post-procedure. Similarly, the secondary outcome measure – postoperative use of IV fentanyl via PCA – also did not show any difference in between the intervention arm vs. the placebo arm. Hence, the previously reported effect of PEMF (as an adjunct intervention) on postoperative pain intensity and analgesia use in other surgical settings cannot be verified. These findings imply that adjunct PEMF use for postoperative pain management may not be recommended at this juncture given the current state of (inconsistent) evidence. Further efforts to understand the reasons of the inconsistent results remain critical.

A review of the existing evidence pool suggested several potential reasons for the inconsistent results. Firstly, a notable feature among the existing evidence on PEMF and its effect on postoperative pain is their small sample sizes in general. Besides the study reported by Stocchero et al. (2014) [15], the study sizes of those described above range from 11 to 72 with the median study size being 36. There are two problems with studies of small sample sizes. A small study may risk not being able to detect a clinically significant difference due to the lack of statistical power, hence committing a type II error [22]. This is a well-known limitation when a study with a small sample size is reported, particularly when the finding is negative. A more important but less well-appreciated problem with a small study size is related to a phenomenon known as the “small study effect” [23]. It has been found that trials with a small number of patients are more likely to report larger and more significant treatment effects compared to their larger counterparts. One of the possible reasons, as illustrated by Kjaergard and colleagues [24] is the lower study quality often associated with small studies. The lack of adequate sequence generating, allocation concealment and blinding can significantly exaggerate the intervention effect compared to larger trials.

Secondly, trials with subjective outcome measures, such as pain score in this context, also have been reported to have the potential of exaggerated effect sizes especially if concealment or blinding is inadequate. This is in contrast to trials with objective outcome measures such as mortality, as there was little evidence that inadequate concealment and lack of/ineffective blinding would significantly distort the estimated effect sizes. Furthermore, the difference in the subjective experience of pain may also result in large variations in patient-reported VAS pain scores and their need of pain medications. As reported by Svaerdborg and colleagues [18] in their study, some patients reported a VAS score of 9 to 10 during the first postoperative day and while others reported VAS scores of only 1, despite having the same procedure and main management regime. As a result, the risk of registering a PEMF effect on postoperative pain can (potentially) be larger (hence, biased) in pain studies of a small sample size.

Lastly, there is also a possibility that biological and clinical factors that influence postoperative pain outcomes might also affect the efficacy of PEMF. For instance, different surgical conditions and related procedures might bring about a different mix of pain aetiologies, thereby affecting the effect of PEMF on cellular mechanisms that induces pain during the acute postoperative period. Hence giving rise to the possibility that PEMF efficacy might differ in different surgical conditions and procedures. Biologically, genetic factors are known to play a significant role in influencing one’s responses to pain and analgesia [25, 26]. The production of opioid metabolizing enzymes and expression of various pain receptors and signal transduction elements are affected by various genetic factors. It also interacts with other physio-pathological, psychological as well as environmental factors that give rise to various pain-related outcomes [27]. These differences are known to give rise to the large variability in terms of pain perception and postoperative pain management needs. Note that this study was conducted on a sample of south-east Asians who may pose very different genetic make-ups compared to the Caucasian population, where most of the earlier studies was focused on [16, 18, 28].

Findings from this study nevertheless should be interpreted with the following limitations. First, different PEMF devices may have slightly different settings. The differences may affect the efficacy and contribute to the inconsistent findings. The potential effect of the different device settings on effectiveness has been previously demonstrated [13]. This study used a device closely resemble that used in previous studies [16, 29] which has obtained a significant positive outcome. Next, we only collected data that spanned over the 12-h observation period during the immediate postoperative phase. There is a possibility that the effect of PEMF will require longer observation to be noticed. Thus, the conclusion of the lack of effect can only be interpreted within the time limit of the data. It is however worth noting that previously reported positive studies [16] have hinted to a potential effect visible as early as 4 h postoperation. Thirdly, in order to balance between the generalizability of the study conclusion and the study efficiency (and safety), we have excluded the younger as well as the older age patients (i.e. < 19 and > 50 years old) despite their significant representation of the population with appendicitis and appendicectomy. Furthermore, we have also excluded those with complicated appendicectomy (which their operative time has exceeded 3 h) and those with potentially altered pain perception (i.e. with underlying chronic pain or long-term use of opioids). These exclusions from the study population have limited the external validity of the study findings and conclusion. Interpretation of this study’s results should therefore consider this limited generalizability. Lastly, this study used the inexpensive SNOSE method for treatment assignment, which is known to have several shortfalls [30, 31]. Because of the lack of accessibility to a more robust treatment allocation system at the time of this study, we were limited to SNOSE as the only practical method available. Given the awareness of the potential bias of using the SNOSE method, the study team has maximized the methodological rigour, especially in terms of the preparation and execution of the procedure. For example, the random sequence was generated, and the envelopes were prepared by a trial researcher not involved in any subsequent trial activities; the officer who perform subject enrolment was independent of the officer who subsequently opened the allocation envelop and perform the device placement.

In conclusion, this study did not verify the effect of PEMF on postoperative pain previously reported. Given the currently available evidence, a recommendation to using PEMF as a routine part of postoperative pain management is not warranted especially for pain management during the immediate postoperative period and of use for short durations. Future larger-scale study may be required to provide more convincing evidence regarding the effect of these devices. A high-quality systematic analysis of the currently available evidence might be required to clarify the current evidence gap in order to better inform future study design and focus.

## Supplementary Information

**Additional file 1: Supplementary material 1.** Logical framework of the study hypothesis.

**Additional file 2: Supplementary Material 2.** AUC for pain score comparison with Wilcoxon rank sum test - results across all the 20 imputed datasets.

**Additional file 3: Supplementary Material 3.** Sensitivity analysis using the best-case-worst-case approach.

**Additional file 4: Supplementary Material 4.** Comparison of 12-hour total fentanyl use (AUC)  results across all the 20 imputed datasets.

**Additional file 5: Supplementary Material 5.** Best-case-worst-case analysis for 12-hour total fentanyl use.

**Additional file 6: Supplementary Material 6.** Annotated version of ***Figure 2.***

## Data Availability

Datasets analysed for the study are available from the corresponding author on reasonable request.
