# Supplementary figures and images for: Pulsed electromagnetic fields for post-appendicectomy pain management: a randomized, placebo-controlled trial
Source: Trials. 2022 Oct 14;23:874. doi: 10.1186/s13063-022-06810-y (PMC9569093; doi:10.1186/s13063-022-06810-y)

**Supplementary material:** Logical framework of study hypothesis

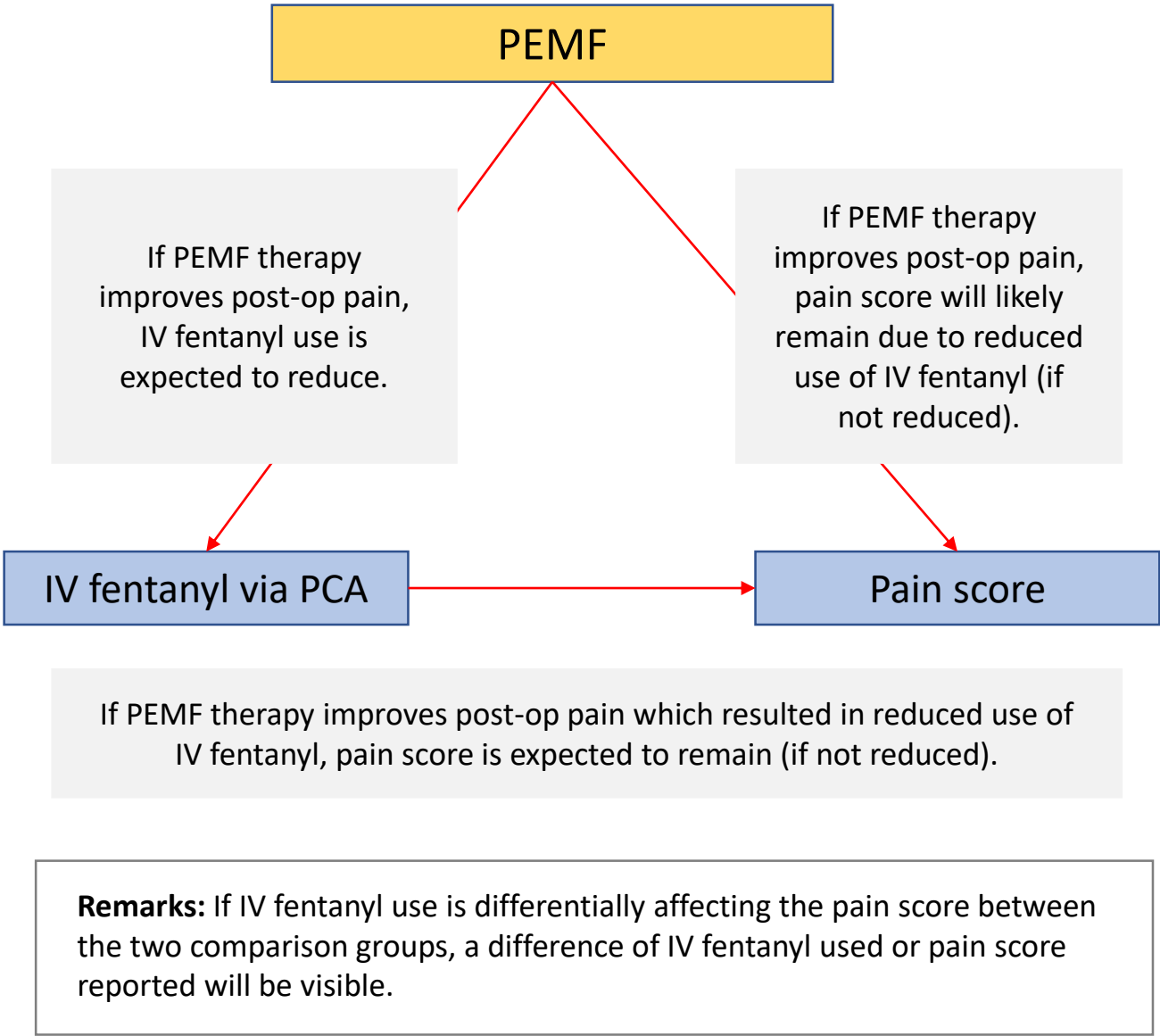

Supplement: Supplementary file 1 — Additional file 1: Supplementary material 1. Logical framework of the study hypothesis. [file 13063_2022_6810_MOESM1_ESM.pdf]
