# Supplementary material for: Pulsed electromagnetic fields for post-appendicectomy pain management: a randomized, placebo-controlled trial
Source: Trials. 2022 Oct 14;23:874. doi: 10.1186/s13063-022-06810-y (PMC9569093; doi:10.1186/s13063-022-06810-y)
Supplement: Supplementary file 2 — Additional file 2: Supplementary Material 2. AUC for pain score comparison with Wilcoxon rank sum test - results across all the 20 imputed datasets. [file 13063_2022_6810_MOESM2_ESM.docx]

**Supplementary Material 2**

Comparison of the AUC for pain score (at rest and on movement) between the PEMF vs. placebo arm using the Wilcoxon rank sum test (W = 1832.5 ~ 1933.0, p-value 0.6192 ~ 0.2985 for resting pain score comparison; W = 1737.0 ~ 1804.5, p-value 0.9892 ~ 0.7296 for movement pain score comparison).

1. **Resting pain score**


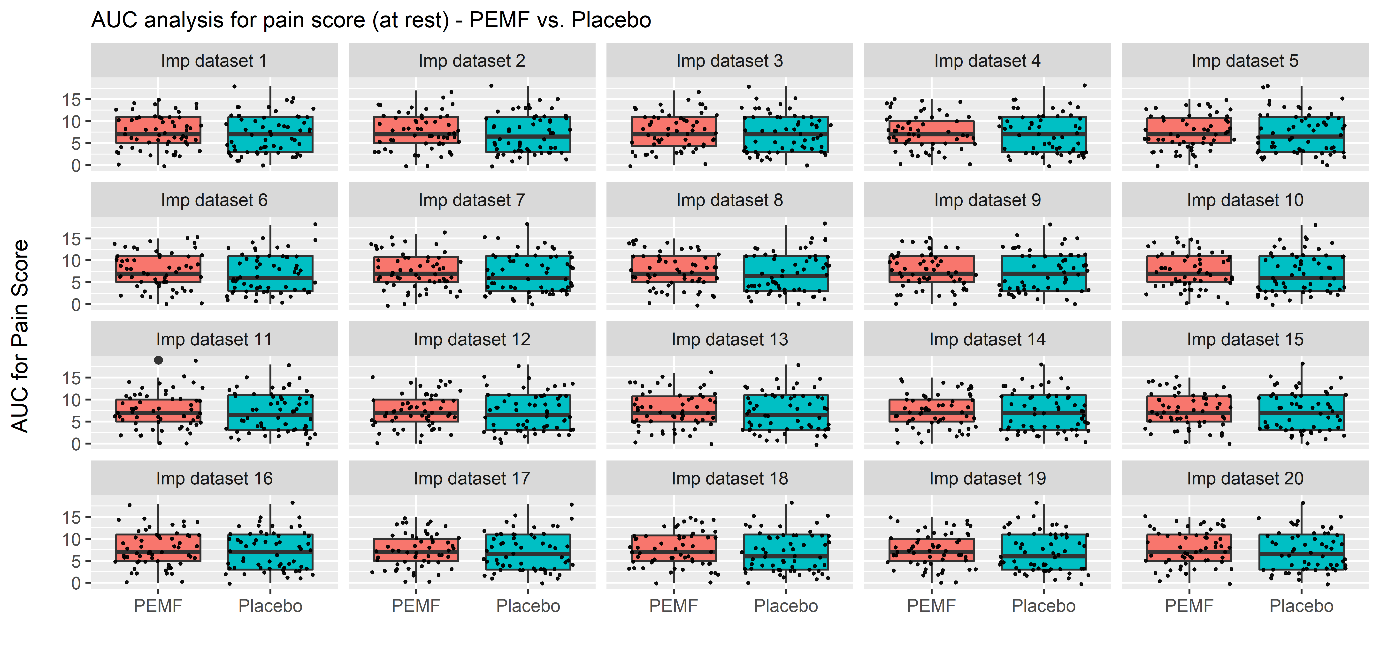


| **Imputed dataset** | **W** | **p-value** |
| --- | --- | --- |
| 1 | 1853.5 | 0.541584735 |
| 2 | 1880.5 | 0.449625370 |
| 3 | 1847.0 | 0.565173916 |
| 4 | 1859.0 | 0.522218039 |
| 5 | 1842.5 | 0.581671103 |
| 6 | 1933.0 | 0.298521107 |
| 7 | 1902.0 | 0.383111335 |
| 8 | 1887.5 | 0.427370802 |
| 9 | 1832.5 | 0.619243405 |
| 10 | 1886.5 | 0.430471578 |
| 11 | 1870.0 | 0.484409038 |
| 12 | 1844.5 | 0.574293183 |
| 13 | 1870.0 | 0.484356856 |
| 14 | 1839.0 | 0.594769121 |
| 15 | 1864.5 | 0.503075461 |
| 16 | 1859.5 | 0.520421131 |
| 17 | 1858.5 | 0.523946067 |
| 18 | 1917.5 | 0.339230433 |
| 19 | 1888.0 | 0.425578475 |
| 20 | 1898.0 | 0.395021448 |

1. Moving pain score


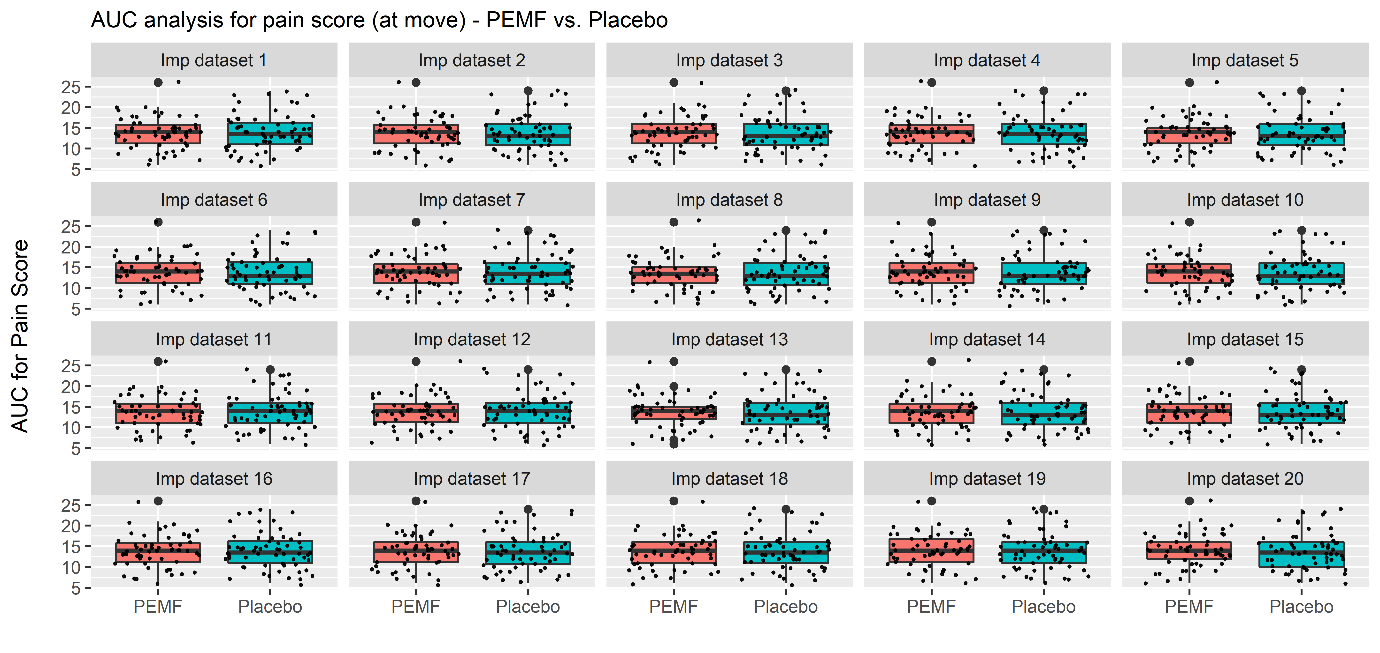


| **Imputed dataset** | **W** | **p-value** |
| --- | --- | --- |
| 1 | 1737.0 | 0.9892203 |
| 2 | 1763.5 | 0.9010955 |
| 3 | 1807.0 | 0.7194276 |
| 4 | 1752.0 | 0.9504242 |
| 5 | 1793.5 | 0.7745501 |
| 6 | 1775.0 | 0.8521589 |
| 7 | 1749.5 | 0.9612152 |
| 8 | 1770.0 | 0.8732924 |
| 9 | 1775.5 | 0.8499757 |
| 10 | 1747.5 | 0.9698227 |
| 11 | 1717.5 | 0.9053854 |
| 12 | 1686.0 | 0.7725105 |
| 13 | 1776.0 | 0.8477998 |
| 14 | 1756.5 | 0.9311147 |
| 15 | 1770.5 | 0.8712154 |
| 16 | 1752.5 | 0.9482952 |
| 17 | 1772.5 | 0.8627753 |
| 18 | 1747.0 | 0.9719812 |
| 19 | 1747.5 | 0.9698434 |
| 20 | 1804.5 | 0.7295724 |
