# Supplementary material for: Pulsed electromagnetic fields for post-appendicectomy pain management: a randomized, placebo-controlled trial
Source: Trials. 2022 Oct 14;23:874. doi: 10.1186/s13063-022-06810-y (PMC9569093; doi:10.1186/s13063-022-06810-y)

**Supplementary Material 3**

Sensitivity analysis using the best-case-worst-case approach


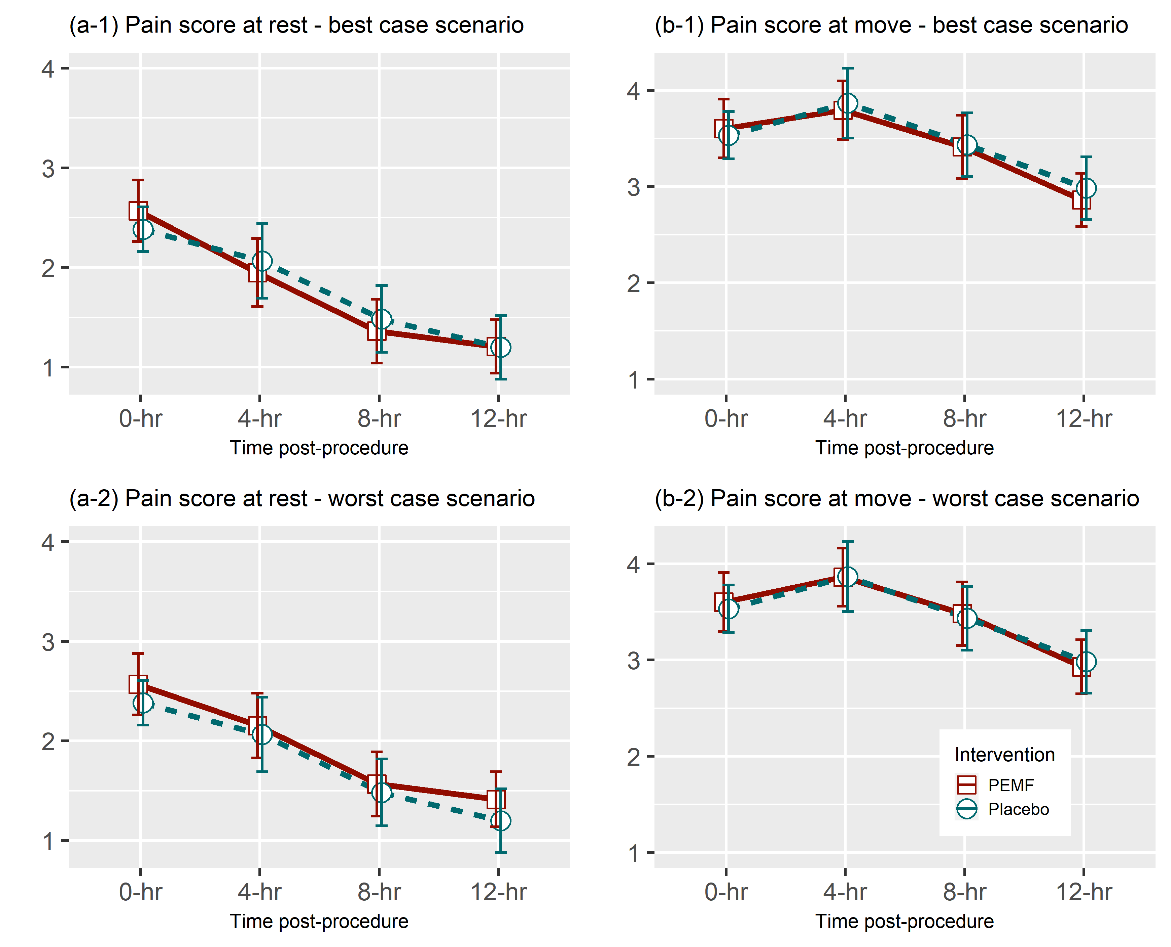


1. **AUC for pain score at rest – Best case vs. Worst case**


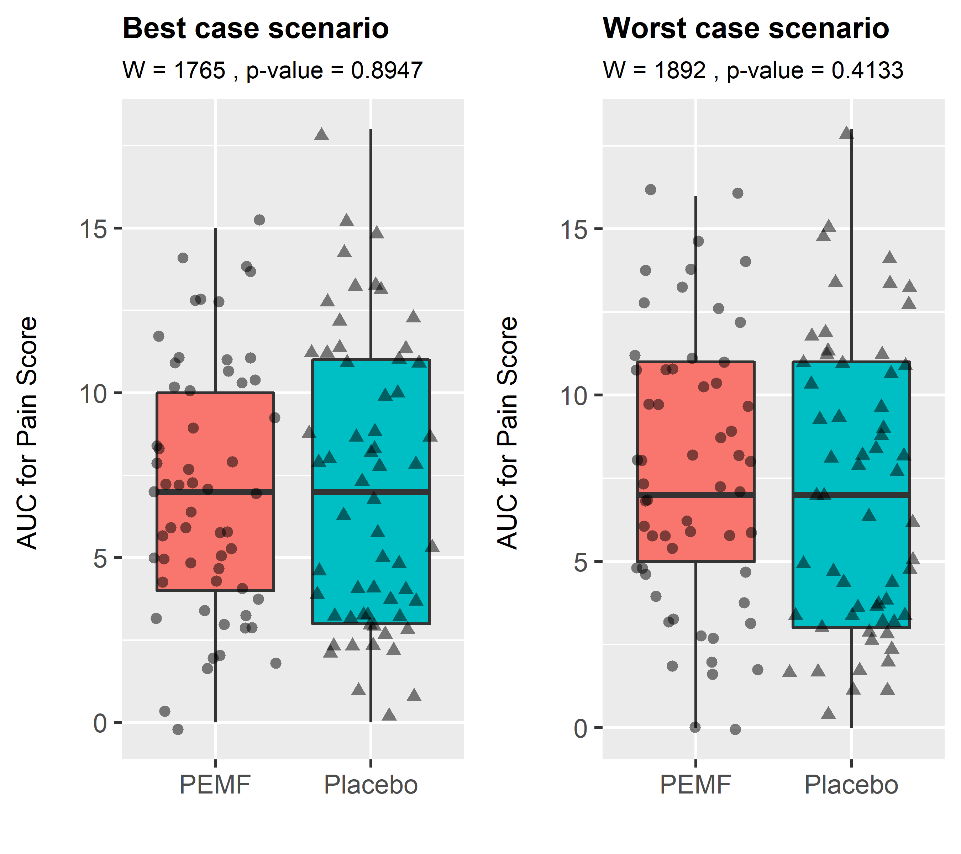


1. **AUC for pain score on movement – Best case vs. Worst case**


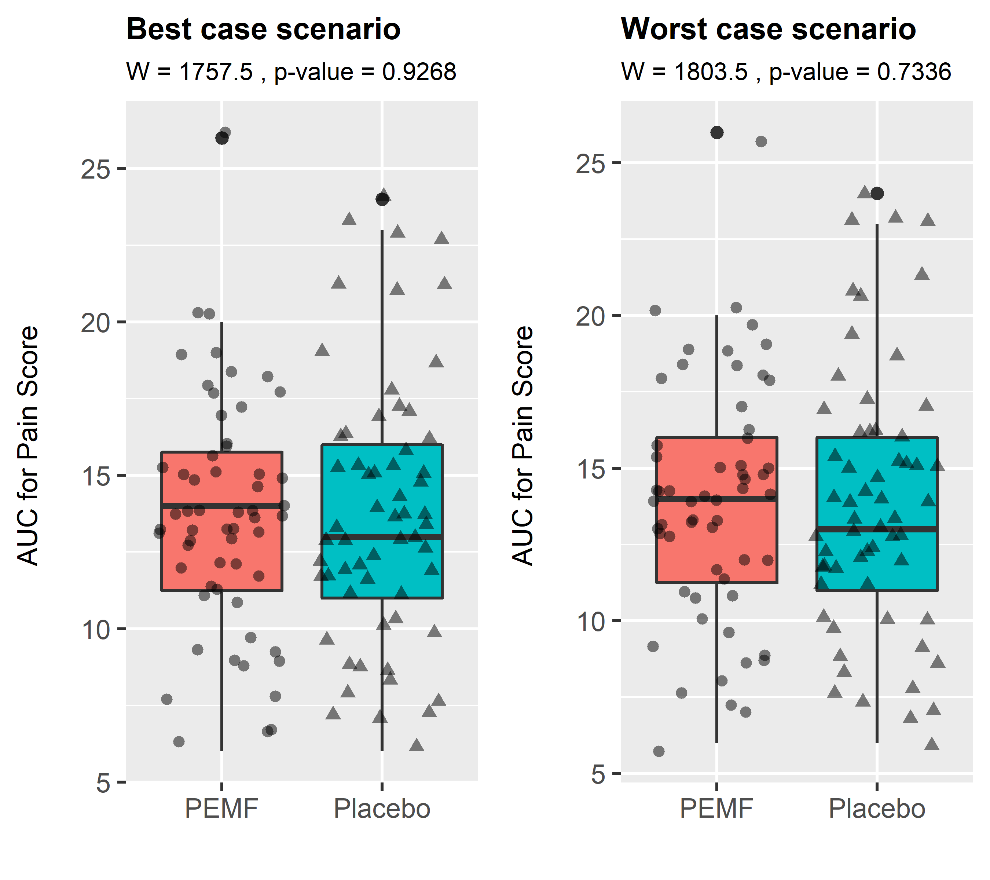

Supplement: Supplementary file 3 — Additional file 3: Supplementary Material 3. Sensitivity analysis using the best-case-worst-case approach. [file 13063_2022_6810_MOESM3_ESM.docx]
