# Supplementary material for: Pulsed electromagnetic fields for post-appendicectomy pain management: a randomized, placebo-controlled trial
Source: Trials. 2022 Oct 14;23:874. doi: 10.1186/s13063-022-06810-y (PMC9569093; doi:10.1186/s13063-022-06810-y)
Supplement: Supplementary file 4 — Additional file 4: Supplementary Material 4. Comparison of 12-hour total fentanyl use (AUC) results across all the 20 imputed datasets. [file 13063_2022_6810_MOESM4_ESM.docx]

**Supplementary Material 4**

Comparison of 12-hour total fentanyl use (AUC) between the PEMF vs. placebo arm across all the 20 imputed datasets


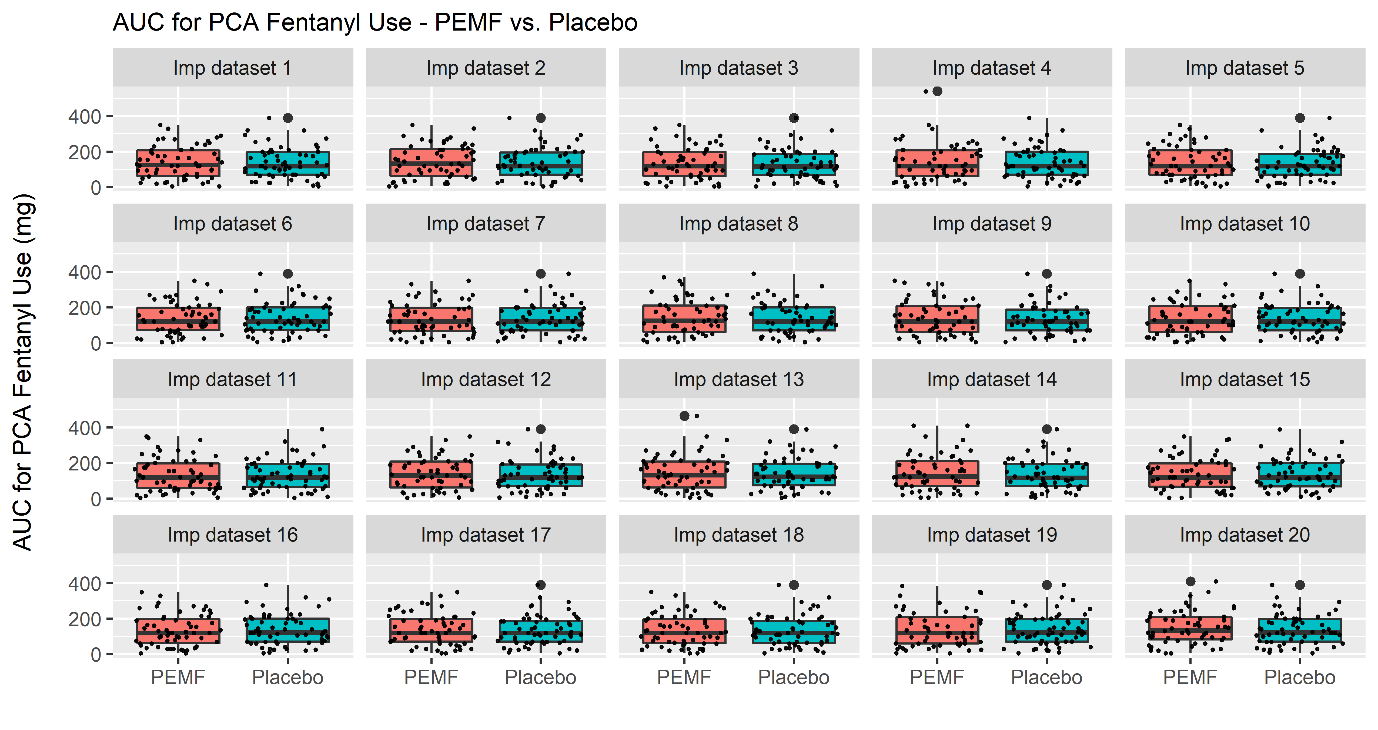


| **Imputed dataset** | **W** | **p-value** |
| --- | --- | --- |
| W | 1765 | 0.895031465 |
| W1 | 1818.5 | 0.674473854 |
| W2 | 1783.5 | 0.816874261 |
| W3 | 1755.5 | 0.93561812 |
| W4 | 1750 | 0.959199228 |
| W5 | 1691.5 | 0.796036992 |
| W6 | 1724 | 0.933480473 |
| W7 | 1780 | 0.831550689 |
| W8 | 1740 | 1 |
| W9 | 1709 | 0.869528052 |
| W10 | 1727 | 0.946333748 |
| W11 | 1808.5 | 0.71422697 |
| W12 | 1752.5 | 0.948476974 |
| W13 | 1859 | 0.523402593 |
| W14 | 1770 | 0.873785811 |
| W15 | 1681 | 0.7527395 |
| W16 | 1784 | 0.814777021 |
| W17 | 1767.5 | 0.884397418 |
| W18 | 1676.5 | 0.734395875 |
| W19 | 1819.5 | 0.670535532 |
