# Supplementary material for: Pulsed electromagnetic fields for post-appendicectomy pain management: a randomized, placebo-controlled trial
Source: Trials. 2022 Oct 14;23:874. doi: 10.1186/s13063-022-06810-y (PMC9569093; doi:10.1186/s13063-022-06810-y)

**Supplementary Material 5**

Best-case-worst-case analysis for 12-hour total fentanyl use


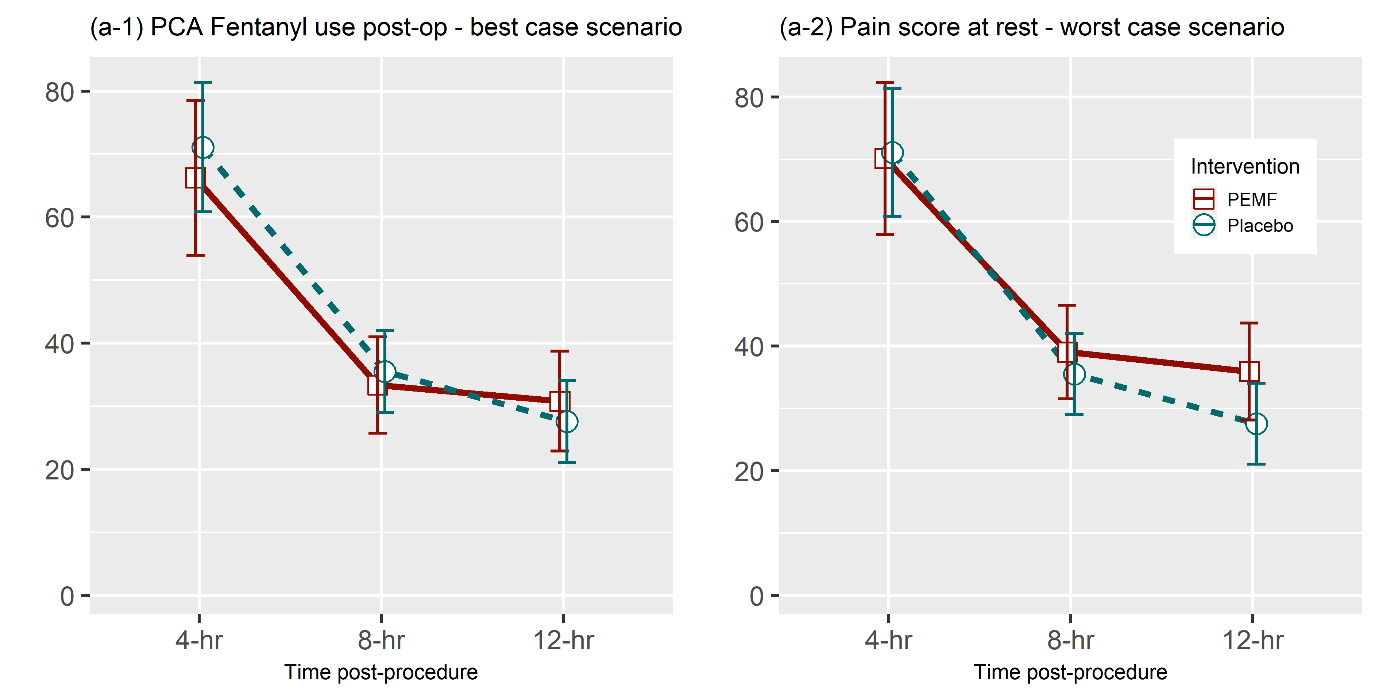


AUC for 12-hour fentanyl use – Best case vs. Worst case


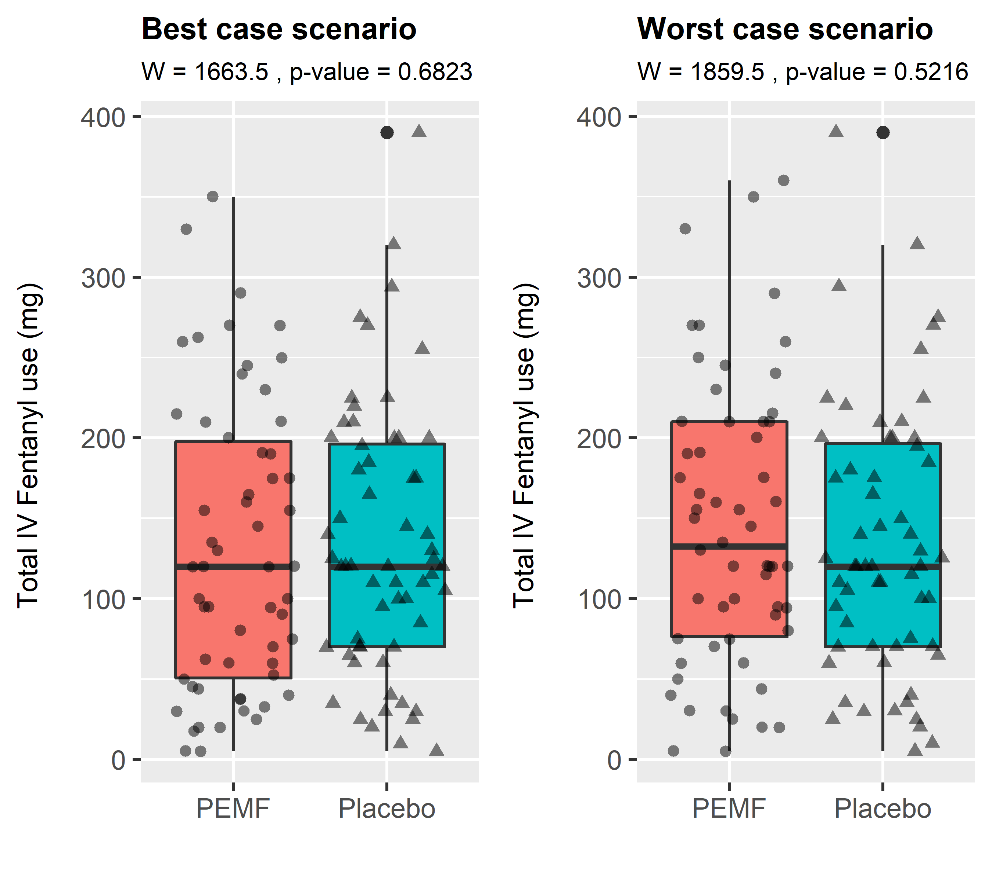

Supplement: Supplementary file 5 — Additional file 5: Supplementary Material 5. Best-case-worst-case analysis for 12-hour total fentanyl use. [file 13063_2022_6810_MOESM5_ESM.docx]
