# Supplementary material for: Pulsed electromagnetic fields for post-appendicectomy pain management: a randomized, placebo-controlled trial
Source: Trials. 2022 Oct 14;23:874. doi: 10.1186/s13063-022-06810-y (PMC9569093; doi:10.1186/s13063-022-06810-y)
Supplement: Supplementary file 6 — Additional file 6: Supplementary Material 6. Annotated version of Figure 2. [file 13063_2022_6810_MOESM6_ESM.docx]

**Supplementary Material 6**

Annotated version of ***Figure 2.*** Changes of Pain Score Post-appendectomy – PEMF (n=58) vs Control (n=60) - which depict the number of subjects with complete data at each observed time point by intervention group.

**Note:** Numerical values shown in the text boxes represent the number of subjects with complete data at each time point by intervention group.


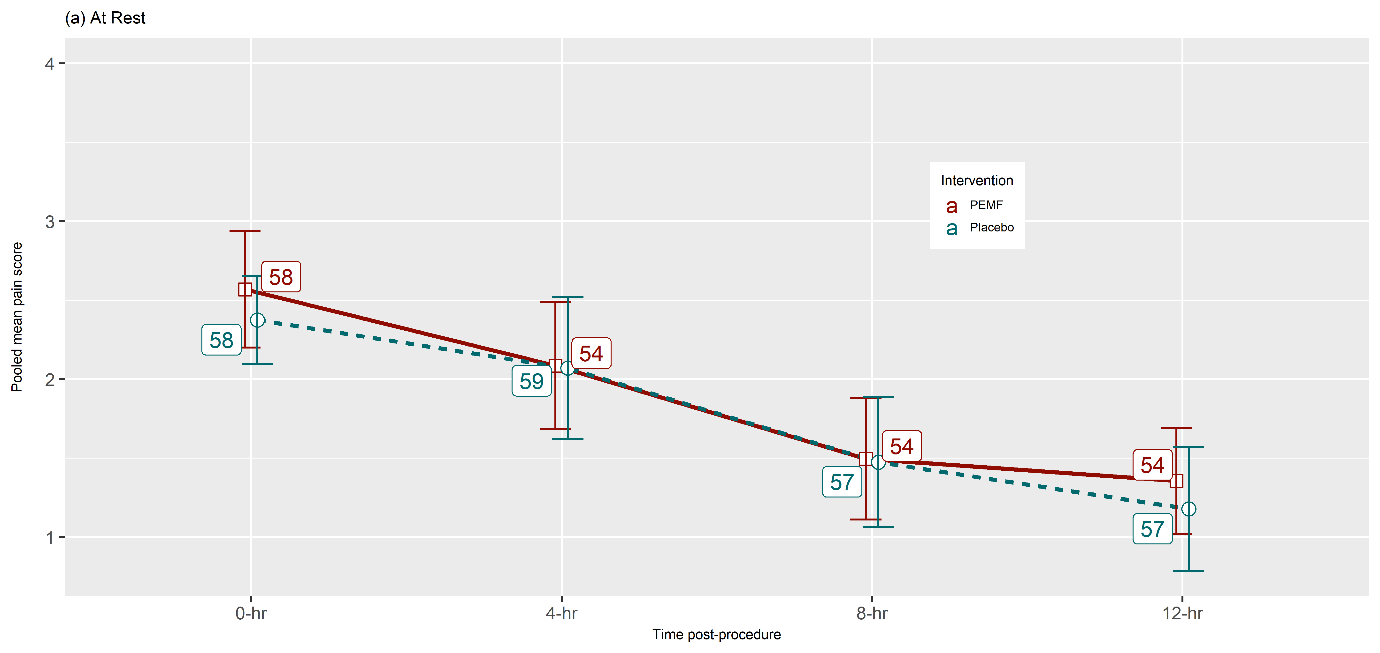


**Note:** Numerical values shown in the text boxes represent the number of subject with complete data at each time point by intervention group.


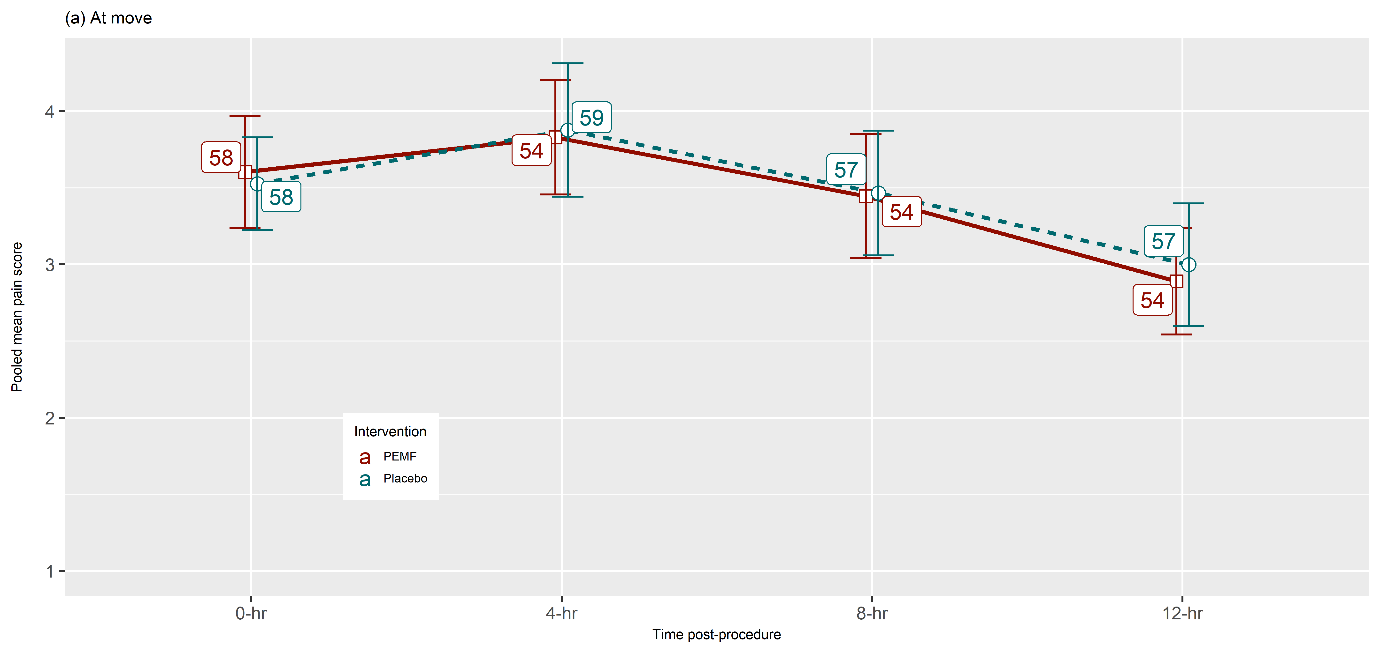


Annotated version of ***Figure 3.*** Changes of PCA Fentanly Use Post-appendectomy – PEMF (n=58) vs Control (n=60) - which depict the number of subjects with complete data at each observed time point by intervention group.

**Note:** Numerical values shown in the text boxes represent the number of subject with complete data at each time point by intervention group.

**
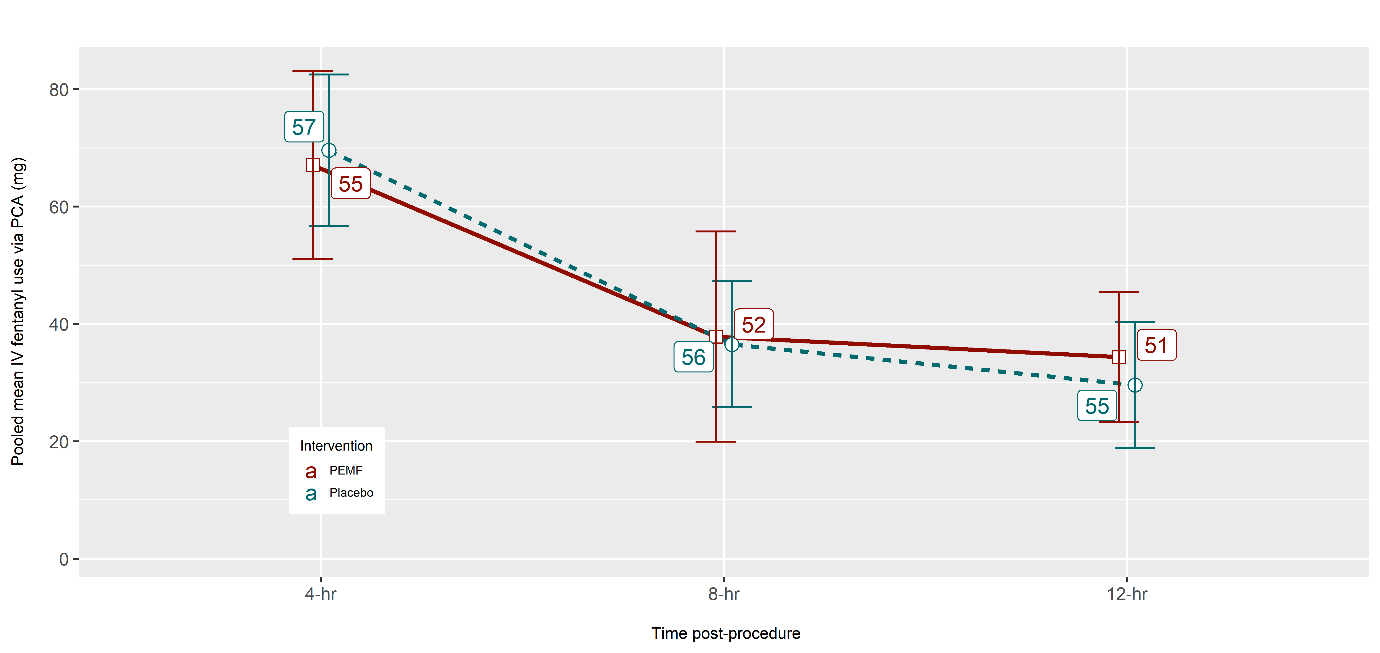
**
